# Umbilical Cord-Derived Mesenchymal Stromal Cells Contribute to Neuroprotection in Neonatal Cortical Neurons Damaged by Oxygen-Glucose Deprivation

**DOI:** 10.3389/fneur.2018.00466

**Published:** 2018-06-15

**Authors:** Takeo Mukai, Arinobu Tojo, Tokiko Nagamura-Inoue

**Affiliations:** ^1^Division of Molecular of Therapy, Center for Advanced Medical Research, Institute of Medical Science, University of Tokyo, Tokyo, Japan; ^2^Department of Cell Processing and Transfusion, Institute of Medical Science, University of Tokyo, Tokyo, Japan

**Keywords:** mesenchymal stromal cell, umbilical cord, neonatal encephalopathy, cerebral palsy, brain derived neurotrophic factor, hepatocyte growth factor

## Abstract

Several studies have reported that human umbilical cord-derived mesenchymal stromal cells (UC-MSCs) restore neurological damage *in vivo* through their secretion of paracrine factors. We previously found that UC-MSCs attenuate brain injury by secreting neurotrophic factors, such as brain-derived neurotrophic factor (BDNF) and hepatocyte growth factor (HGF). However, how these factors contribute to neuroprotection remains unknown. In this study, we aimed to investigate to what extent UC-MSC-derived HGF and BDNF contribute to neuroprotection using a Transwell co-culture system of neonatal cortical neurons damaged by oxygen-glucose deprivation. The influence of HGF and BDNF were determined by investigating neurons in both the presence and absence of UC-MSCs as these cells consistently secrete both factors and can be blocked by neutralizing antibodies. In the co-culture, UC-MSCs significantly improved neuronal injury, as indicated by an increase in immature neuron number, neurite outgrowth, and cell proliferation. Co-culture of damaged neurons with UC-MSCs also exhibited a reduction in the number of neurons displaying signs of apoptosis/necrosis. The neuroprotective actions of UC-MSCs were partially reverted by neutralizing antibodies. Together, our findings reveal that UC-MSC-secreted HGF and BDNF have neuroprotective effects on damaged neurons. Further studies should address the existence of other potential neurotrophic paracrine factors.

## Introduction

Mesenchymal stromal cells (MSCs) can be isolated from several sources, including bone marrow, cord blood, adipose tissue, the placenta, and the umbilical cord (UC) (1–5). Among various sources of MSCs, we focused on the UC for the following reasons: (1) abundant sources and ease of collection, storage, and transport; (2) no invasive process of collection; (3) little ethical controversy; (4) multipotency to differentiate into various cell types; (5) low immunogenicity with significant immunosuppressive ability; and (6) migration ability toward injured sites (5). Two major mechanisms have been postulated for the observed improvements following MSC treatment; namely, anti-inflammatory and neurotrophic mechanisms. UC-MSCs have been reported to exert anti-inflammatory effects via contact with activated T cells and partly through indoleamine 2, 3-dioxygenase and prostaglandin E2 (6, 7). Indeed, it is thought that UC-MSCs exert anti-inflammatory actions on brain lesions in the acute stages of injury. On the other hand, the mechanisms which underlie the neurotrophic effects of UC-MSCs have not been fully elucidated.

We previously reported that intravenously administered UC-derived MSCs (UC-MSCs) attenuate intraventricular hemorrhage-induced injuries, and brain-derived neurotrophic factor (BDNF) and hepatocyte growth factor (HGF) concentration were elevated in serum and cerebrospinal fluid in some part of UC-MSCs administered mice (8). Therefore we aim to confirm the function of BDNF and HGF *in vitro* in this study.

Guo et al. (9) reported the paracrine effects of UC-MSCs on nerve regeneration, observing that UC-MSCs express neurotrophic factors and that UC-MSC-conditioned medium enhances Schwann cell viability and proliferation via increases in nerve growth factor and BDNF expression. However, since HGF and BDNF are not separately inhibited, there is a possibility that each individual of HGF or BDNF may not contribute to neurotrophic effect. In addition, neuroprotective effect, such as anti-apoptosis/necrosis effect, was not examined.

In this study, we focused on whether BDNF and HGF secreted by UC-MSCs exert neuroprotective effect in addition to the neurorestorative effect *in vitro*, and verified our previous results.

## Materials and methods

### UC-MSC preparation

This study was carried out in accordance with the recommendations of Ethics Committee of the Institute of Medical Science, the University of Tokyo, and the NTT Medical Center Hospital and Yamaguchi Hospital, Japan with written informed consent from all subjects. All subjects gave written informed consent in accordance with the Declaration of Helsinki. The protocol was approved by the Ethics Committee of the Institute of Medical Science, the University of Tokyo, and the NTT Medical Center Hospital and Yamaguchi Hospital, Japan. UC-MSCs were isolated from three individual donors using previously reported methods (8, 10). Briefly, the UCs were collected after informed consent was obtained from pregnant women planning to undergo cesarean sections. Frozen-thawed UC tissues were minced into fragments and underwent the improved explant culture method (11). Tissue fragments were cultured with α-minimal essential medium (αMEM; Wako Pure Chemical Industries, Ltd., Japan) supplemented with 10% fetal bovine serum and antibiotics-antimycotics (Antibiotic-Antimycotic, 100X; Life Technologies, USA) at 37°C with 5% CO_2_. Fibroblast-like adherent cells that migrated from the UC tissue fragments were harvested using TrypLE Select (Life Technologies), and were defined as passage 1 UC-MSCs. The harvested cells underwent four passages, after which they were used for experimental analyses as well as previous report (8). UC-MSCs were preserved in cryoprotectant and thawed before use.

### Cortical neuron primary cultures

All experiments were carried out in accordance with the Animal Experiment Committee of the Institute of Medical Science, the University of Tokyo. Cortical neurons from B6 Albino mice (B6N-Tyrc-Brd/BrdCrCrl, Charles River Laboratories International, Inc.) were prepared according to previous reports (12, 13). Briefly, embryonic day 16 fetuses (*n* = 21) were taken from euthanized, pregnant mice in sterile conditions. Fetal brains were removed and cortical tissues were dissected under a microscope. The meninges were then removed and cortical tissues were chopped into small pieces. Cells were dispersed followed by mechanical trituration using Neuron Dissociation Solutions (Wako Pure Chemical Industries, Ltd., Japan) and filtered through a 70 μm pore-size cell strainer. Cells were then resuspended in neurobasal medium (GIBCO) supplemented with 2% B27 (Invitrogen) and plated onto Poly-L-Lysine Culture Dishes (BioCoat™, Corning Inc. Japan). Cells were cultured in a humidified incubator at 37°C with 5% CO_2_, and half of the medium was replaced with fresh solution every 3 days. To reduce contamination by glial cells, 10 μM cytosine arabinofuranoside (Sigma-Aldrich) was added for 24 h on the 4th day of culture. We cultured cortical neurons for 7days, and OGD procedure was performed.

### Oxygen-glucose deprived neurons co-cultured with UC-MSCs

A model of neonatal cortical neurons injured by OGD was established as previously described (13, 14). For deprivation of glucose, primary cortical neurons were washed twice with phosphate-buffered saline (PBS) and cultured in glucose-free Dulbecco modified eagle medium (GIBCO). Cells were incubated in an anaerobic chamber (95% N_2_, 5% CO_2_) (ASTEC Co, Ltd., Japan) at 37°C. The OGD condition was maintained for 4 h, after which cells were re-oxygenated in the original medium and placed in a normoxic chamber (37°C, 5% CO_2_). After injury by OGD was completed, co-culture with UC-MSCs was started immediately. Neurons were co-cultured with UC-MSCs according to previously reported methods (10). Briefly, using a 24-well trans-well chamber (Corning, USA) equipped with an 8-μm filter membrane, cortical neurons were cultured in the bottom chamber, while UC-MSCs were plated in the upper chamber at 5 × 10^4^ cells/well overnight for 24 h at 37°C with 5% CO_2_. In experiments aimed to measure HGF and BDNF concentrations in the culture supernatant, αMEM without fetal bovine serum was used during co-culture.

### Multiplex flow cytometric beads assay

For HGF and BDNF inhibition, the following neutralizing antibodies (NAbs) were added to UC-MSC culture media in order to deplete HGF and BDNF, as previously reported (12, 15, 16): anti-HGF neutralizing antibody (ab10678, Abcam) and recombinant human TrkB Fc chimera protein (#688-TK; R&D Systems). Briefly, cells were treated with 0.5 μg/mL of anti-HGF antibody or 1–2 μg/mL recombinant human TrkB Fc chimera protein at plating; media was not changed during the course of the experiment. As a negative control, appropriate recombinant human IgG1 Fc (R&D Systems) was used. In order to measure the neutralized concentration of human-HGF and human-BDNF, the supernatant of UC-MSCs was analyzed using a multiplex flow cytometry beads assay (#111116 and #111362, HQ-Plex Kit; Bay Bioscience, Japan). Also in order to measure mouse-HGF and mouse-BDNF, the supernatant of cortical neurons was analyzed using a multiplex flow cytometry beads assay (#211230 and #211226, HQ-Plex Kit; Bay Bioscience, Japan). All samples were analyzed in triplicate according to the manufacturers' instructions. Bead fluorescence readings were done by a flow cytometry apparatus (BD™ FACS Canto II), and data were analyzed using FCAP Array ver.3.0.1 Software (BD Biosciences, CA, USA). Results are expressed in pg/ml.

### Immunocytochemical assessment of cortical neurons

The expression of neural protein markers and a mitotic marker was analyzed by immunocytochemistry. Briefly, neurons were fixed with 4% paraformaldehyde for 30 min at room temperature, blocked in 5% skim milk and 0.3% Triton-X 100 (Sigma-Aldrich, USA), and incubated overnight with primary antibodies at 4°C. The primary antibodies included mouse anti-human microtubule-associated protein 2 (MAP-2; 1:200 dilution, Abcam), rabbit anti-growth associated protein-43 (GAP-43; 1:500 dilution, Abcam), rabbit anti-BrdU (1:200 dilution, Abcam), and mouse anti-phospho histone H3 (S10) (1:500 dilution, Abcam). Secondary antibodies used were donkey anti-mouse IgG heavy and light chain-specific (H&L) (Alexa Fluor® 488) (1:1,000; Abcam), and donkey anti-rabbit IgG H&L (Alexa Fluor® 594) (1:1,000; Abcam). Nuclei were counterstained with 4',6-diamidino-2-phenylindole (DAPI; Sigma-Aldrich, Japan), and images were acquired using a fluorescence microscope (Nikon Eclipse Ti, Nikon Instruments Inc., Japan) and NIS-Elements microscope imaging software version 4.10 according to previously described imaging methods (10). For BrdU labeling, the cells were incubated in the 10 μM BrdU (ab142567, Abcam) labeling solution for 24 h at 37°C in a CO2 incubator before staining. To objectively enumerate phospho Histone H3- and BrdU-positive cells, Image J software version 1.49 was used. Positive cells were counted in five randomly-selected fields at a magnification of 200x using a microscope (Nikon Eclipse Ti, Nikon Instruments Inc., Japan), and the proportion of positive cells was calculated as the number of histone H3-positive cells/total number of cells × 100%. The length of neurites in each neuron was measured by tracing using Image J (17–20). Neurites were identified by immunofluorescence with MAP-2 and counts were made in at least three randomly selected microscopic fields (containing 50 cells).

### Western blotting

Proteins were extracted from the cells according to the manufacturer instructions as previously described (8, 10). Protein concentrations of the samples were measured using the RC DC Protein Assay kit (Bio-Rad) and equal amounts of the protein and sample loading buffer were boiled for 5 min and separated by sodium dodecylsulfate polyacrylamide gel electrophoresis, followed by transfer onto PVDF membranes (Immobilon-P Membrane, PVDF, Millipore). The membranes were blocked by 5% skim milk in Tris-NaCl-Tween buffer and incubated overnight at 4°C with the primary antibodies, GAP-43 (mentioned above) and anti-beta-tubulin (Wako Pure Chemical Industries, Ltd., Japan), at the recommended dilutions. This was followed by incubation with secondary antibodies (horseradish peroxidase-conjugated anti-rabbit IgG or horseradish peroxidase-conjugated anti-mouse IgG) for 1 h at room temperature. PVDF membranes were imaged using an enhanced chemiluminescence system with Pierce ECL Western Blotting Substrate (Thermo Scientific).

### Cortical neuron proliferation assay

To evaluate whether co-culture with UC-MSC attenuated the death of cortical neurons after OGD, cell proliferation was determined using the Cell Proliferation ELISA kit, BrdU (Roche, #11669915001) according to the manufacturer's instructions. Briefly, neuronal BrdU incorporation was quantitatively evaluated using OD450 reader measurements (Bio-Rad iMark Microplate Absorbance Reader Version 1.02.01).

### Fluorochrome-labeled inhibitor of caspases (FLICA)

To evaluate caspase activity in cortical neurons after OGD, cells were incubated in fluorochrome-labeled inhibitor of caspases (FLICA) solution (Immunochemistry Technologies, FAM-FLICA® Poly Caspase Assay Kit) at 37°C for 1 h. After three washes, cells were labeled with propidium iodide (PI) and immediately imaged using a fluorescence microscope. Cell nuclei were counter-stained with Hoechst 33342. PI labeling was used with FLICA to identify four populations of cells: living (FLICA–, PI–); early apoptotic (FLICA+, PI–); late apoptotic (FLICA+, PI+); and necrotic (FLICA–, PI+). Unstained live cells were labeled with only Hoechst 33342, and necrotic cell membranes appeared compromised and stained with PI in red. Cells in the late apoptotic phase were dually stained with FAM-FLICA (green) and PI, and cells in the early apoptotic stage were stained only with FAM-FLICA. Apoptotic/necrotic cells were counted using a microscope (Nikon Eclipse Ti, Nikon Instruments Inc., Japan), and the proportion of apoptotic/necrotic cells /total number of cells ×100% was calculated (containing total number of cells ranging from 321 to 556 cells).

### Statistical analysis

Values are expressed as mean ± standard deviation (SD) from three independent experiments. Differences between groups were analyzed with JMP 10.0.2 software (SAS Institute, USA). Groups were compared using one-way analyses of variance (ANOVAs), followed by Turkey's tests. *P*-values of 0.05 or less were regarded as statistically significant.

## Results

### Constitutive secretion of HGF and BDNF from UC-MSCs co-cultured with cortical neurons after OGD

**Figure 1:**
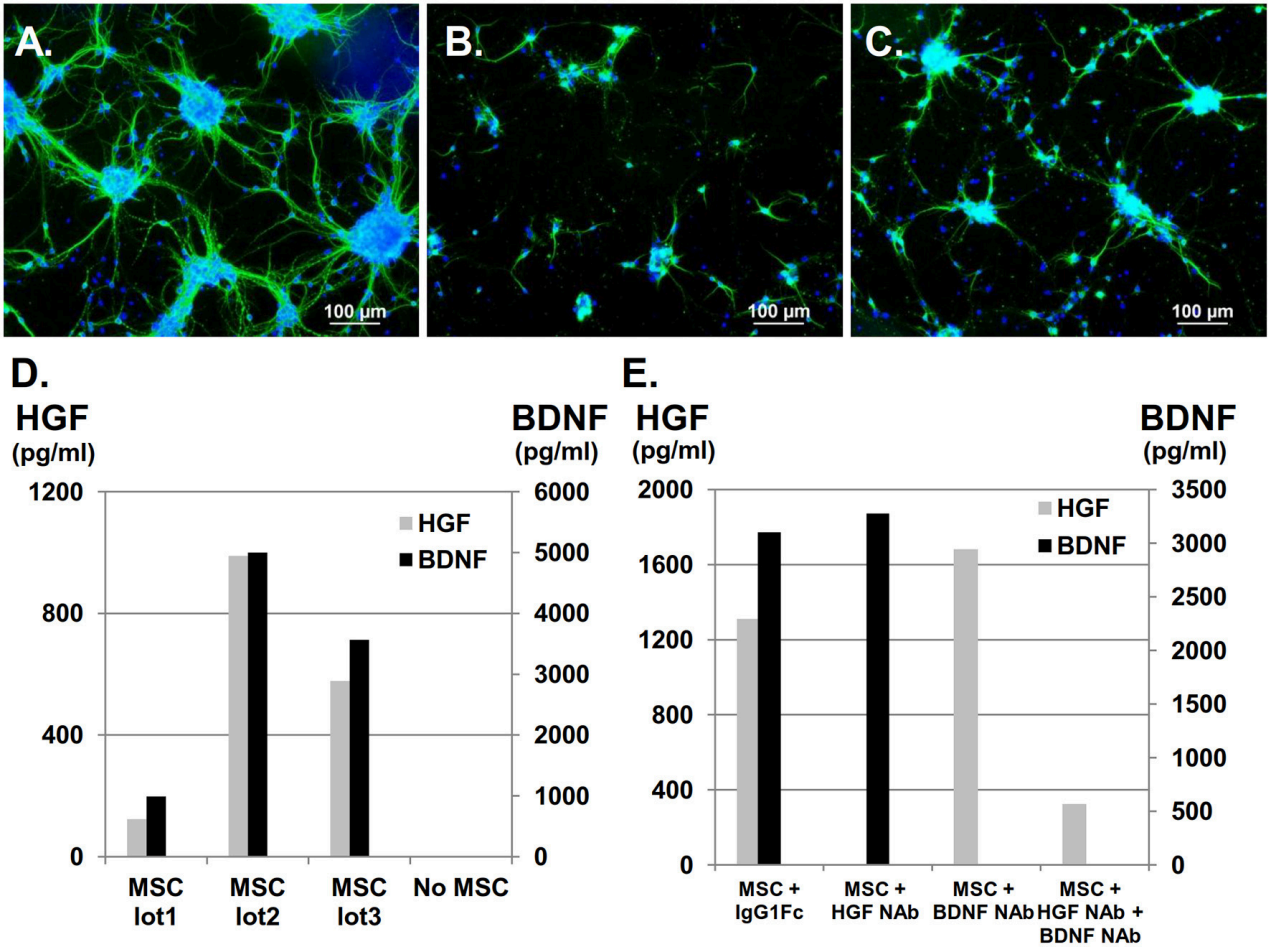
Constitutive secretion of HGF and BDNF from UC-MSCs co-cultured with cortical neurons after OGD. UC-MSCs co-cultured with cortical neurons post-OGD and stained with MAP-2. Mature neurons are stained in green and nuclei are counterstained with DAPI in blue. **(A)** Control, **(B)** OGD, **(C)** OGD + MSC (Scale bar = 100 μm). **(D)** UC-MSC-secreted HGF and BDNF. **(E)** HGF NAb and recombinant Human TrkB Fc chimera were used for the inhibition of HGF and BDNF, respectively. Recombinant human IgG1 Fc was used as a negative control. NAb, neutralizing antibody.

Primary cultures of neonatal cortical neurons exhibited typical morphology, showing a neurite outgrowth-forming network (Figure 1A) that could be visualized by positive MAP-2 labeling in green, which represented mature neurons. Neurites and neuronal clusters disappeared after OGD (Figure 1B), but co-culture with UC-MSCs restored mature neurons, long neurites, and cluster formations (Figure 1C). Using a multiplex flow cytometry bead assay to analyze HGF and BDNF concentrations in medium containing UC-MSCs confirmed that both factors were constitutively secreted from UC-MSCs, and that their concentrations varied by UC-MSC lot (*n* = 3, Figure 1D). Importantly, HGF and BDNF concentrations could be appropriately reduced by the addition of NAbs (Figure 1E). Using these methods, the following experiments were performed: (1) control, (2) neurons injured by OGD (OGD), (3) neurons injured by OGD co-cultured with UC-MSCs (OGD+MSC), (4) OGD + MSC with anti-HGF NAb (OGD+MSC+ HGF NAb, (5) OGD + MSC with anti-BDNF NAb (OGD+MSC+BDNF NAb), and (6) OGD + MSC with both anti-HGF NAb and anti-BDNF NAb (OGD+MSC+ HGF NAb + BDNF NAb).

### UC-MSCs exert neuroprotective effect on cortical neurons after OGD injury

**Figure 2:**
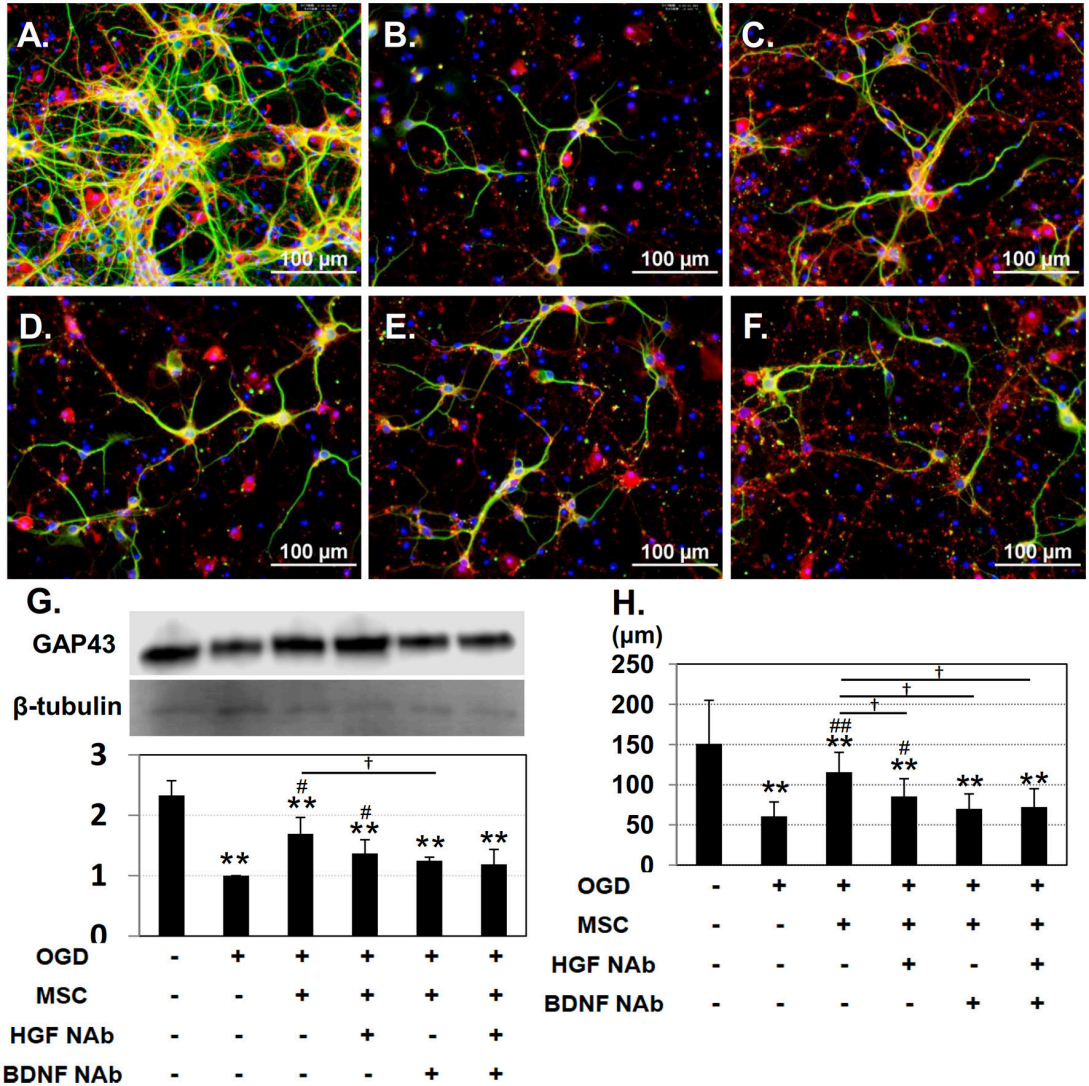
UC-MSCs restore both mature and immature neurons after OGD. Immunostaining showing immature, developing neurons labeled with GAP-43 (red) and mature neurons labeled with MAP-2 (green). **(A)** Control, **(B)** OGD, **(C)** OGD + MSC, **(D)** OGD + MSC + HGF NAb, **(E)** OGD + MSC + BDNF NAb, and **(F)** OGD + MSC + HGF NAb + BDNF NAb (Scale bar = 100 μm). **(G)** Western blotting and quantitative analysis of GAP-43 expression. β-tubulin was used as an internal control. **(H)** Quantitative analysis of neurite outgrowth. The data shown are representative of three independent experiments. ^**^p < 0.01 compared to the control group, ^##^*p* < 0.01, ^#^*p* < 0.05 compared to the OGD group, and ^†^*p* < 0.05 compared to the MSC group. NAb, neutralizing antibody.

Under control conditions, both immature (GAP-43-positive; red) and mature (MAP-2-positive; green) neurons could be observed (Figure 2A). After OGD injury, neurites appeared diminished in length, and the number of cells positive for GAP-43 was reduced (Figure 2B). Co-culture with UC-MSCs maintained the mature neurons with long neurites to some extent, and increased the number of GAP-43-positive immature neurons (Figure 2C). Addition of NAbs reduced the UC-MSC-mediated improvement (Figures 2D–F). Quantitative analysis of GAP-43 expression revealed a significant decrease in the OGD compared to the control group, and that co-culture with UC-MSCs significantly improved GAP-43 expression relative to levels observed in the OGD group. On the other hand, addition of NAbs tended to decrease the expression of GAP-43 (Figure 2G). The length of neurites identified by immunofluorescence with MAP-2 was significantly shortened in the OGD relative to the control group, and co-culture with UC-MSCs significantly improved neurite outgrowth length. The addition of anti-HGF + BDNF NAbs significantly reduced neurite length after OGD (Figure 2H). Interestingly the effects of UC-MSCs on GAP-43 expression and neurite length were reverted partially by the addition of HGF and BDNF NAbs, and there was no synergistic effect by the addition of them.

**Figure 3:**
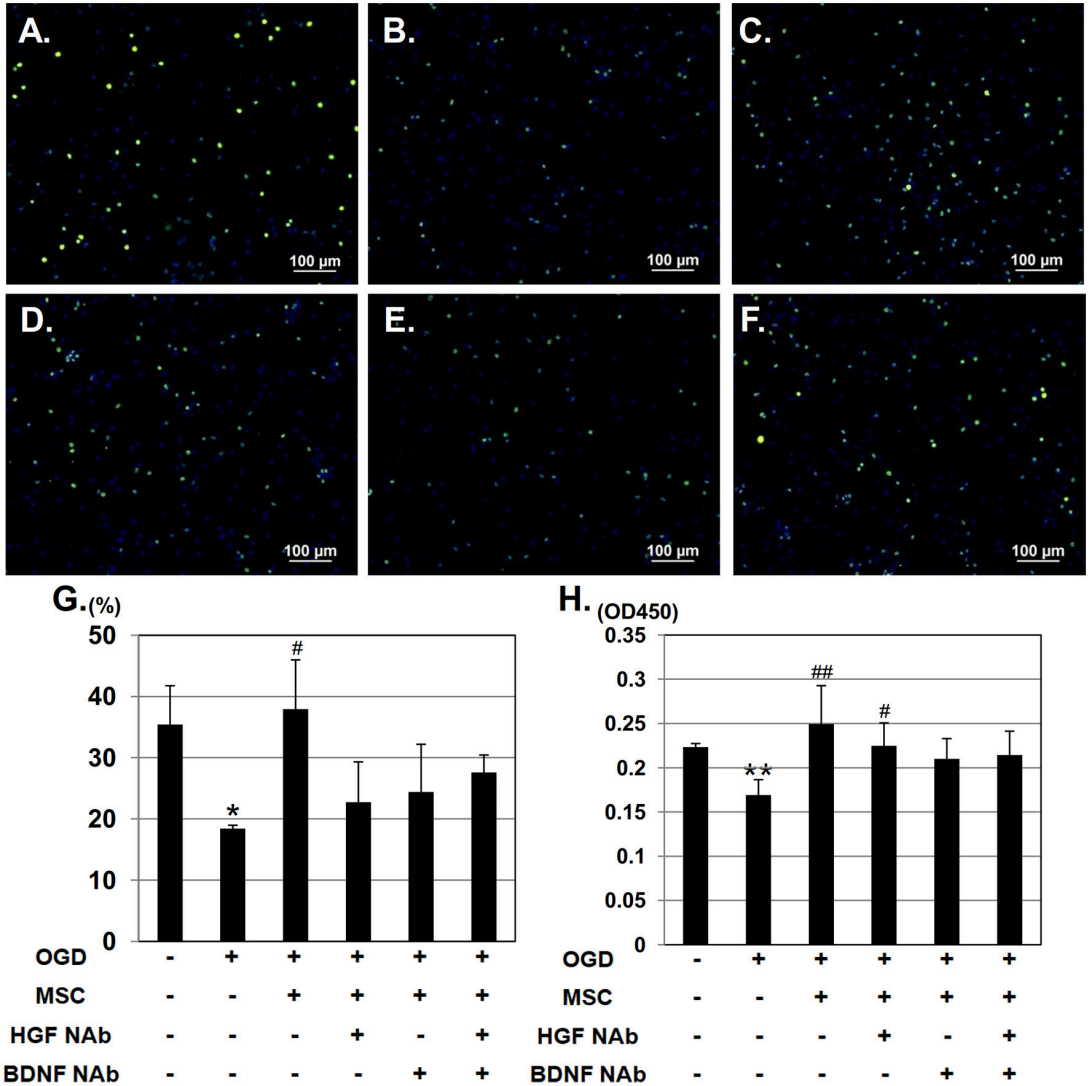
UC-MSCs support neuronal mitosis after OGD. Immunostaining showing mitotically proliferating neurons stained with phospho histone H3 (green) and counterstained with DAPI (blue). **(A)** Control, **(B)** OGD, **(C)** OGD + MSC, **(D)** OGD + MSC + HGF NAb, **(E)** OGD + MSC + BDNF NAb, and **(F)** OGD + MSC + HGF NAb + BDNF NAb (Scale bar = 100 μm). **(G)** Ratio of the number of phospho histone H3-positive cells to the total number of cells. **(H)** BrdU incorporation into proliferating neurons, measured and compared between six groups. ^**^p < 0.01, ^*^p < 0.05 compared to the control group, and ^##^*p* < 0.01, ^#^*p* < 0.05 compared to the OGD group. NAb, neutralizing antibody.

To investigate the effect of HGF and BDNF on neurorestoration, cortical neurons were stained with the mitotic marker, anti-phospho histone H3 and counterstained with DAPI (Figure 3A). In the OGD group, the number of phospho histone H3 - positive cells decreased, whereas this reduction was restored in neurons co-cultured with UC-MSCs (Figures 3B,C). Addition of NAbs, however, attenuated this recovery (Figures 3D–F). Quantitative analysis revealed a significantly higher number of proliferating phospho histone H3 -positive cells were seen in neurons co-cultured with UC-MSCs compared to neurons in the OGD group. whereas addition of NAbs to the OGD+MSC group decreased the amount of mitotic cells (Figure 3G). On the other hand, BrdU incorporation into neurons was reduced significantly in the OGD group. Co-culture with UC-MSCs significantly increased BrdU incorporation post-OGD (Figure 3H); however, the effect was not attenuated by the addition of NAbs. We also performed double staining of phospho histone H3 and BrdU (Supplementary Figure 1). Quantitative analysis revealed a tendency that higher number of proliferating phospho histone H3-positive cells and BrdU-positive cells were seen in neurons co-cultured with UC-MSCs compared to neurons in the OGD group, whereas the effect of NAbs to the OGD+MSC group wasn't observed.

**Figure 4:**
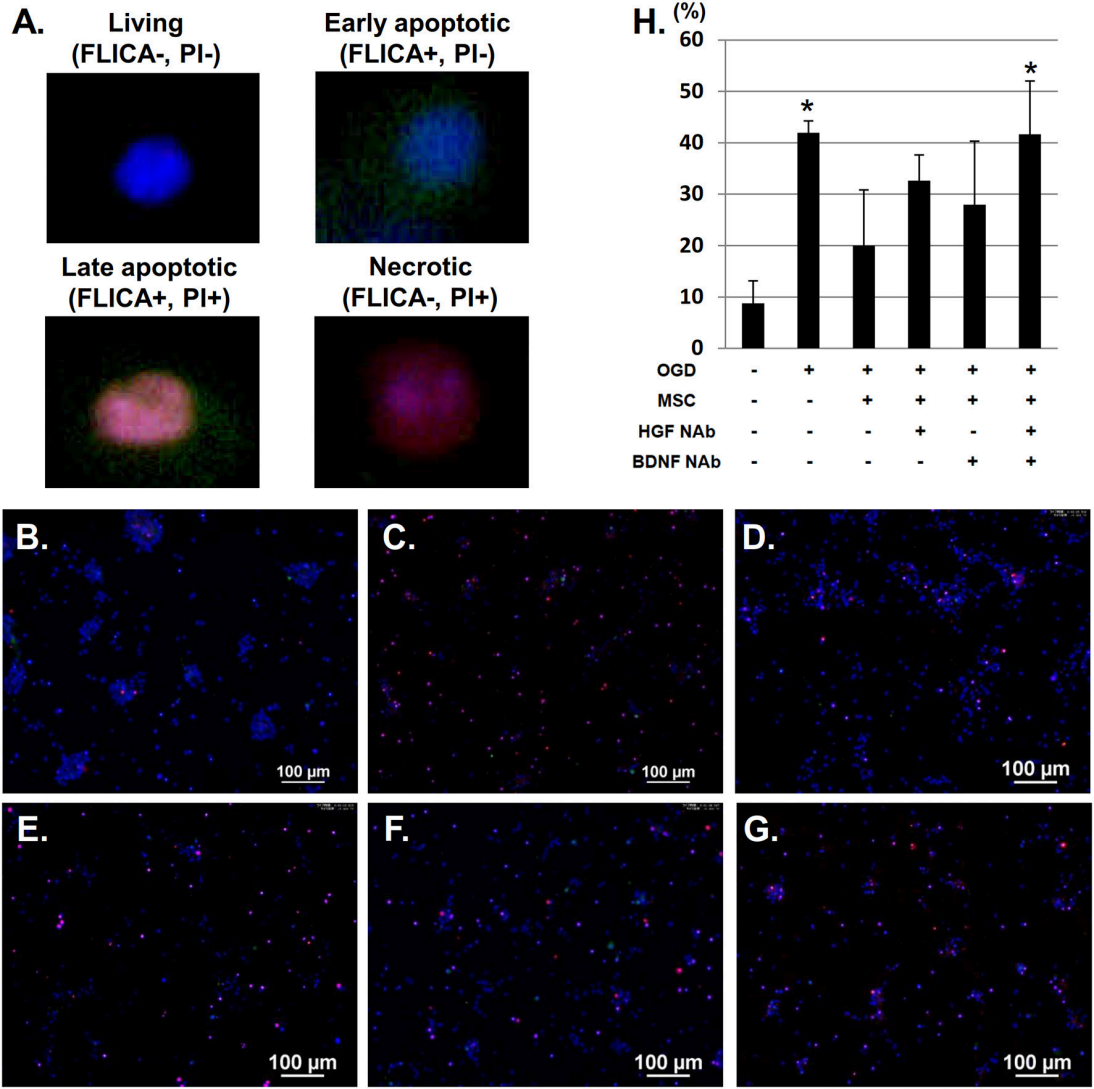
UC-MSCs restore apoptotic and necrotic neurons after OGD. **(A)** FLICA labeling showing the proportion of apoptotic and necrotic neurons after OGD. Four populations of cells can be identified: living (FLICA–, PI–); early apoptotic (FLICA+, PI–); late apoptotic (FLICA+, PI+); and necrotic (FLICA–, PI+). Cell nuclei are counterstained with Hoechst 33342. **(B)** Control, **(C)** OGD, **(D)** OGD + MSC, **(E)** OGD + MSC + HGF NAb, **(F)** OGD + MSC + BDNF NAb, and **(G)** OGD + MSC + HGF NAb + BDNF NAb (Scale bar = 100 μm). **(H)** Ratio of apoptotic and necrotic cells to the total number of cells. ^*^p < 0.05 compared to the control group. NAb, neutralizing antibody.

Next, we examined the neuroprotective effect of HGF and BDNF on neuronal apoptosis/necrosis after OGD using FLICA labeling. We identified four populations of cells: living (FLICA–, PI–); early apoptotic (FLICA+, PI–); late apoptotic (FLICA+, PI+); and necrotic (FLICA–, PI+) (Figures 4A–G). Quantitative analysis of the ratio of apoptotic and necrotic cells to total cells revealed significantly more apoptotic/necrotic cells in the OGD group compared to the control group. This analysis also demonstrated that co-culture with UC-MSCs reduced the number of cortical neurons displaying signs of apoptosis and necrosis post-OGD. This improvement was attenuated by the addition of anti-BDNF and anti-HGF NAbs (Figure 4H).

## Discussion

Recently, UC-MSCs have attracted attention for their potential in treating neurological disorders, as several studies using neurological disease models have reported improvements after UC-MSC transplantation, and clinical studies using UC-MSCs to treat traumatic brain injury and cerebral palsy have already been implemented (21–26). The neurotrophic effects of UC-MSCs can be characterized by two mechanisms of action; (1) neurogenic differentiation and cell replacement, and (2) secretion of neurotrophic factors. UC-MSCs can differentiate into neural cells expressing high levels of neural markers (10, 27, 28). However, intravenously administered UC-MSCs in hosts that are not immunocompromised are likely ultimately eliminated. Indeed, our previous study revealed that UC-MSCs injected in neonatal mice with intraventricular hemorrhage are eliminated after 3 weeks. Interestingly, human BDNF and HGF was detected in the serum and cerebrospinal fluid of these mice (8).

Consistent with a previous report (9), we confirmed constitutive secretion of HGF and BDNF from UC-MSCs, which were inhibited by the HGF NAb and recombinant human TrkB Fc chimeras, as described in previous reports (12, 15). However, the concentrations of secreted HGF and BDNF demonstrated variability that was dependent on UC-MSC lot. Lot-to-lot variation in these secreted neurotrophic factors is an important issue considering their potential for clinical application.

In this study, we successfully generated an *in vitro* OGD model of primary cortical -neurons, as indicated by shortened neurites, a reduction in the number of network-forming neurites, fewer developing neurons, decreased cell proliferation, and increased apoptosis/necrosis. Co-culture with UC-MSCs was sufficient to maintain MAP2-positive mature neurons showing extended neurites and cluster formations, whereas HGF and BDNF NAbs significantly attenuated the restorative effect of UC-MSCs on neurite elongation.

Firstly we investigated neurorestorative effect of UC-MSCs using phospho histone H3 and BrdU experiment. Higher number of phospho histone H3 and BrdU-positive mitotic cells were observed in UC-MSC co-cultured cells compared to cells in the OGD group, whereas the reverse effects of HGF and BDNF NAbs on cell mitosis were not significant in histone H3 and BrdU experiments. Next we confirmed neuroprotective effect of UC-MSCs on cortical neurons after OGD injury by apoptosis/necrosis assay. The results demonstrated that co-culture with UC-MSCs reduced the number of cortical neurons displaying signs of apoptosis and necrosis post-OGD and this improvement was attenuated by the addition of anti-BDNF and anti-HGF NAbs.

These results suggest that HGF and BDNF secreted from UC-MSCs may support neuroprotection through anti-apoptotic effect rather than neurorestoration, and that there is the possibility of other UC-MSC-secreted trophic factors.

HGF—a multi-functional growth factor originally reported as a potent mitogen for mature parenchymal hepatocytes in primary culture—plays an important role in tissue regeneration in the nervous system. It has been reported that HGF binds a tyrosine kinase receptor encoded by the human proto-oncogene, c-Met, and that HGF and c-Met are expressed in both the adult and fetal central nervous system (29, 30). Following activation of the tyrosine kinase receptor, the induction of several downstream pathways, such as the phosphatidylinositol 3-kinase/Akt, MAP-kinase, and signal transducers and activators of transcription 3 pathways, lead to neurorestorative, anti-apoptotic and neurogenic effects (29–31). Indeed, Liu et al. (32) reported the neuroprotective effects of UC-MSCs infected with adenovirus-expressing HGF in a model of Parkinson's disease, suggesting that HGF acted via the promotion of damaged cell regeneration. These previous reports, together with findings of the current study, support the important role of HGF in neurogenesis and maintaining cell viability.

Similar to HGF, BDNF (the second neurotrophic factor to be characterized, after NGF and before neurotrophin-3) is also expressed in the adult and developing brain, and plays a key role in the proliferation, survival, and differentiation of neurons (33–35). BDNF binds to the tropomyosin-related kinase family of receptor tyrosine kinases, which activate the phosphatidylinositol 3-kinase/Akt and MAP-kinase pathways. BDNF also binds to the p75 neurotrophin receptor, which activates nuclear factor-kB—a protein complex important in inducing the activation of pro-survival and pro-differentiation genes (36). Consistent with the supportive role of BDNF described above, we found that BDNF inhibition attenuated the extent of neurorestoration observed in cortical neurons co-cultured with UC-MSCs and increased the amount of apoptotic/necrotic cells.

To exclude the possibility of mouse-BDNF and mouse-HGF secreted from damaged cortical neurons in this study, we measured the concentration of mouse-BDNF and mouse-HGF in the supernatant of the cortical neurons with or without OGD. The result showed that mouse-BDNF and mouse-HGF were very low below detection limit (data not shown), but they should be taken into consideration in the present study. In addition, even if it seems more likely that UC-MSCs secrete those neurotrophic factors, we cannot exclude the possibility that UC-MSCs induce self-secretion of BDNF and HGF by injured neurons. These endogenous neurotrophic factors must be considered for future study.

In conclusion, although the presence of other UC-MSC-secreted factors likely exist, the current study showed that UC-MSCs exert their neuroprotective effects partially through secretion of BDNF and HGF by inhibiting the apoptosis/necrosis of injured neurons. Considering that UC-MSCs have been administered to treat several neurological disorders, including cerebral palsy, traumatic brain injury, and hereditary spinocerebellar ataxia (25, 26, 37), the vast potential that UC-MSCs have in the clinic encourage us to facilitate allogeneic third-party UC-MSC therapies for brain injuries.

## Author contributions

All authors listed have made a substantial, direct and intellectual contribution to the work, and approved it for publication.

### Conflict of interest statement

The authors declare that the research was conducted in the absence of any commercial or financial relationships that could be construed as a potential conflict of interest.

## Supplementary material

Supplementary Figure 1 UC-MSCs support neuronal mitosis after OGD. Immunostaining showing mitotically proliferating neurons stained with phospho histone H3 (green), BrdU (red) and counterstained with DAPI (blue). **(A)** Control, **(B)** OGD, **(C)** OGD + MSC, **(D)** OGD + MSC + HGF NAb, **(E)** OGD + MSC + BDNF NAb, and **(F)** OGD + MSC + HGF NAb + BDNF NAb (Scale bar = 100 μm). **(G)** Ratio of the number of phospho histone H3-positive cells and BrdU-positive cells to the total number of cells. ^**^*p* < 0.01, ^*^*p* < 0.05 compared to the control group. NAb, neutralizing antibody.

## Abbreviations

UC: umbilical cord
MSCs: mesenchymal stromal cells
OGD: oxygen - glucose deprivation
MAP-2: microtubule-associated protein 2
GAP-43: growth associated protein 43
BDNF: brain-derived neurotrophic factor
HGF: hepatocyte growth factor
DAPI: 4'6-diamidino-2-phenylindole
NAb: neutralizing antibody
FLICA: fluorescent-labeled inhibitor of caspases.
