# Supplementary figures and images for: Umbilical Cord-Derived Mesenchymal Stromal Cells Contribute to Neuroprotection in Neonatal Cortical Neurons Damaged by Oxygen-Glucose Deprivation
Source: Front Neurol. 2018 Jun 15;9:466. doi: 10.3389/fneur.2018.00466 (PMC6013549; doi:10.3389/fneur.2018.00466)

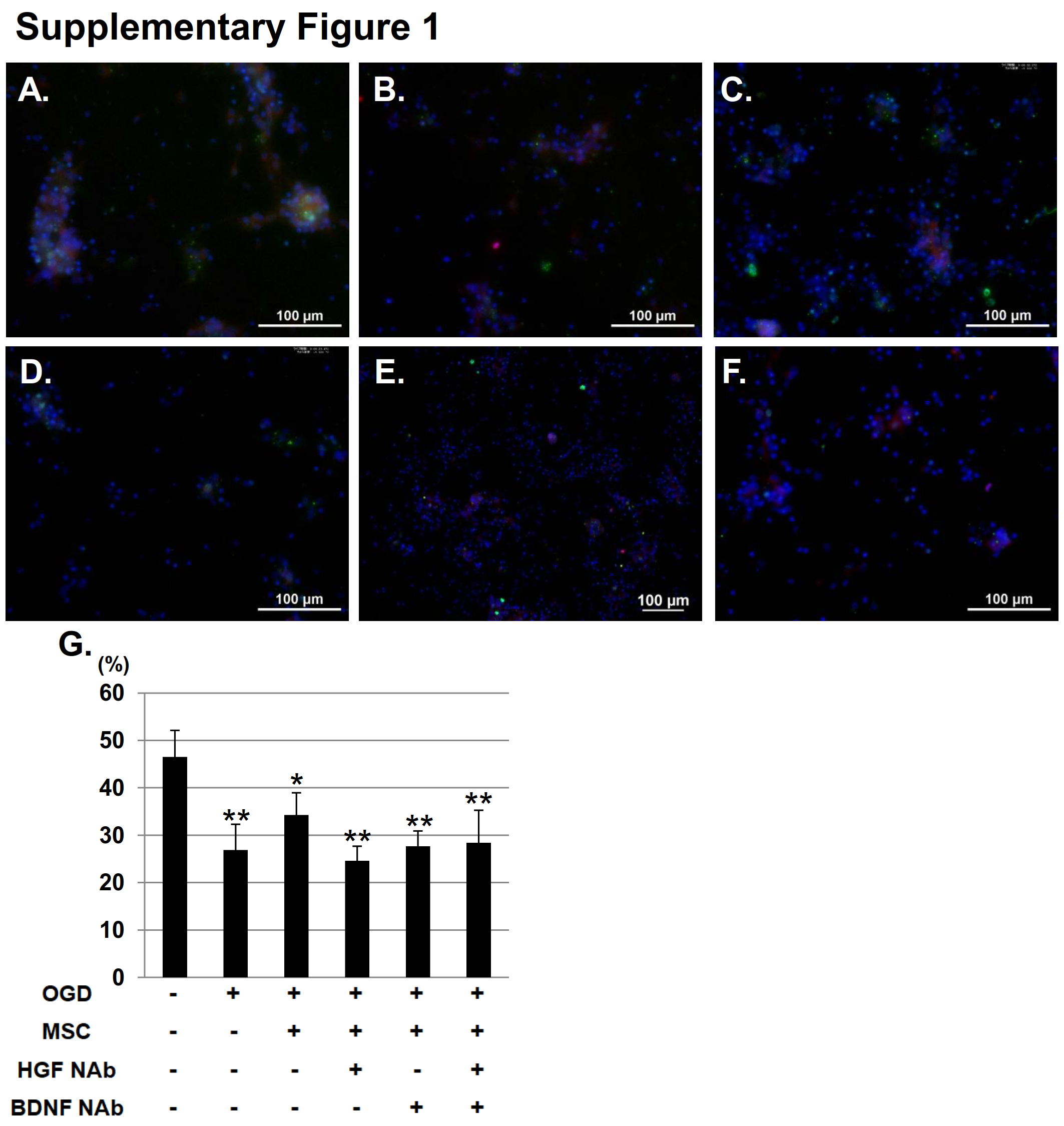

Supplement: Supplementary Figure 1 — UC-MSCs support neuronal mitosis after OGD. Immunostaining showing mitotically proliferating neurons stained with phospho histone H3 (green), BrdU (red) and counterstained with DAPI (blue). (A) Control, (B) OGD, (C) OGD + MSC, (D) OGD + MSC + HGF NAb, (E) OGD + MSC + BDNF NAb, and (F) OGD + MSC + HGF NAb + BDNF NAb (Scale bar = 100 μm). (G) Ratio of the number of phospho histone H3-positive cells and BrdU-positive cells to the total number of cells. **p < 0.01, *p < 0.05 compared to the control group. NAb, neutralizing antibody. [file Image_1.TIF]
